# A Review for Detecting Gene-Gene Interactions Using Machine Learning Methods in Genetic Epidemiology

**DOI:** 10.1155/2013/432375

**Published:** 2013-10-21

**Authors:** Ching Lee Koo, Mei Jing Liew, Mohd Saberi Mohamad, Abdul Hakim Mohamed Salleh

**Affiliations:** Artificial Intelligence and Bioinformatics Research Group, Faculty of Computing, Universiti Teknologi Malaysia, Skudai, 81310 Johor, Malaysia

## Abstract

Recently, the greatest statistical computational challenge in genetic epidemiology is to identify and characterize the genes that interact with other genes and environment factors that bring the effect on complex multifactorial disease. These gene-gene interactions are also denoted as epitasis in which this phenomenon cannot be solved by traditional statistical method due to the high dimensionality of the data and the occurrence of multiple polymorphism. Hence, there are several machine learning methods to solve such problems by identifying such susceptibility gene which are neural networks (NNs), support vector machine (SVM), and random forests (RFs) in such common and multifactorial disease. This paper gives an overview on machine learning methods, describing the methodology of each machine learning methods and its application in detecting gene-gene and gene-environment interactions. Lastly, this paper discussed each machine learning method and presents the strengths and weaknesses of each machine learning method in detecting gene-gene interactions in complex human disease.

## 1. Introduction

Genome-wide association studies (GWAS) had offered various kinds of techniques to study DNA variations that are associated with human diseases. As a result, single nucleotide polymorphisms (SNPs) had been widely used in GWAS to unravel genetic basis by testing individual variants that are associated with complex human diseases. The knowledge of SNPs in GWAS is particularly important because genes can influence human disease, and many genetic landscape of human disease is still unknown and uncharacterized. Thus, the knowledge on the effect of SNPs on common disease is needed in order to understand the variation of genetic underlying human diseases that rises through gene-gene and gene-environment factors. Hence, the interactions between gene-gene and gene-environment are particularly important to discover the genetic architecture underlying genetic disease.

**Figure 1:**
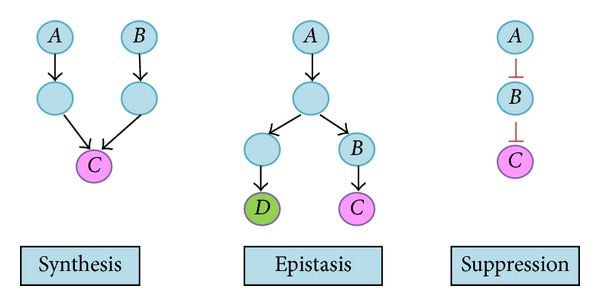
Types of gene-gene interactions [1].

The term gene-gene interactions is also known as epistasis and genetic interactions. It also can be defined as a logical interaction between two or more genes that affects the phenotype of organisms. The ultimate goals of gene-gene interactions are to recognize gene functions, identify pathways and discover potential drug targets. Moreover, there are various types of gene-gene interactions which are synthetic-interaction, epistatic interaction, and suppressive-interaction which are shown in Figure 1. These interactions are particularly important due to the effect of a gene on individual phenotype is depending on more than one additional genes.

As shown in Figure 1, there are various types of gene-gene interactions. For instances, synthetic-interaction between two genes is that genes *A* and *B* are on different parallel pathways that can obtain the purple phenotype *C*. If either of the genes is knockout, the purple phenotype *C* still can be viewed. However, if both of the genes are knockout, it will result in a nonpurple phenotype. Next, the example of epistatic-interaction that is the wild type holds a mixed purple and green phenotype of genes *C* and *D*. A gene knockout of gene *B* cannot obtain a purple phenotype of gene *C*, but green phenotype of gene *D* still can be seen. A gene knockout of gene *A* cannot obtain the green and purple phenotypes. Furthermore, the example of suppressive-interaction is wild type phenotype showing a purple phenotype since gene *A* suppresses gene *B* and gene *C* is active. A gene knockout of gene *B* has no effect for result purple phenotype. A knockout of gene *A* results in a nonpurple phenotype since gene *B* is still suppressing gene *C* and if both of the genes *A* and *B* are knockout will result in wild type phenotype.

Moreover, there are various challenges that are associated with gene-gene interactions and needed to be addressed. The greatest challenge is the increasing volume of data that needed to be analysed. The number of potential interactions increases as the number of SNPs increases. This leads to high computational complexity because it needs to enumerate all possible SNP combinations in multilocus associations at genome wide scale. Hence, jointly analysing such SNP combinations by high throughput genotyping technologies is also one of the challenges faced in genome wide association studies. Besides, the existence of high dimensionality of data and multiple polymorphisms has also increases the computational complexity of traditional statistical approaches to analyse large scale genetic data. Hence, the existence of machine learning methods can overcome these challenges because machine learning methods are flexible in recognizing the gene-gene interactions that can contribute to individual's disease status. 

According to [2], *learning* can be defined as “to gain knowledge, or understanding of, or skill in, by study, instruction, or experience” and “modification of a behavioral tendency by experience.” Thus, machine learning can be defined as a computer learns from experience through algorithms. In this review, we focus on supervised machine learning in which the machine undergoes learning process and predicts the type of gene interactions based on the given inputs. Hence, the goal of supervised machine learning is based on given input variables and then predicts the output variables [3]. The methods of machine learning that we focus on in this review are neural networks (NNs), support vector machine (SVM), and random forests (RFs).

## 2. Neural Networks

**Figure 2:**
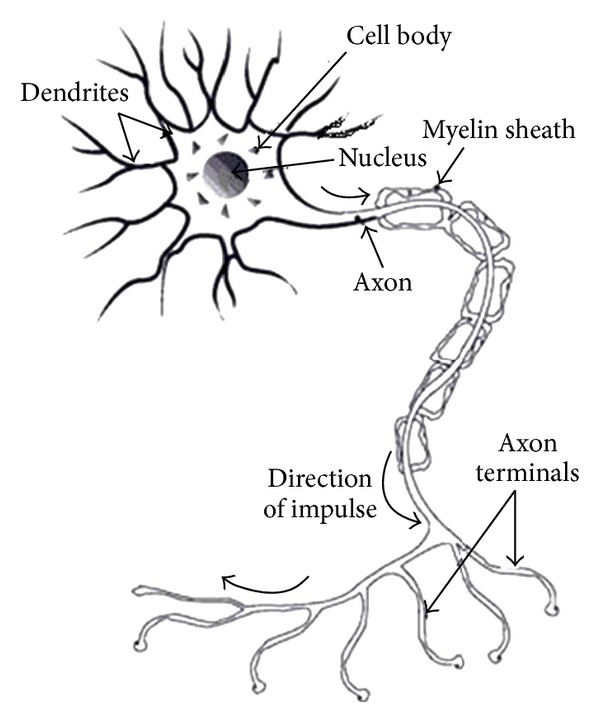
Structure of biological neuron.

According to [4], neural networks (also known as artificial neural networks) were established based on imitation of the neurons on how they work in the brain. Basically, the neuron receives responses from the environment, and once the input exceeds critical level, the neuron transmits the signal to another neuron through the axons.  Figure 2 shows the structure of biological neuron.

According to [5], the corresponding artificial features from the structure of the biological neurons are as follows.

- Dendrites represent inputs in the neural network.
- Soma (cell body) represents artificial neuron which is the summation and thresholding part of the model.
- Axon represents weighted inputs of artificial neuron.

According to [4], neural networks are suitable for modelling human genetics studies due to the ability of handling large quantities of data with reasonable computation time as the scalability of data increases exponentially. Moreover, neural networks are able to approximate any type of genetic etiology that underlies phenotypic values because neural networks are universal approximators. Neural networks are model free so no assumptions should be made about genetic architecture that produce in a particular phenotype. This property is particularly important when mining the high dimensional data.

### 2.1. Methodology of Neural Networks

**Figure 3:**
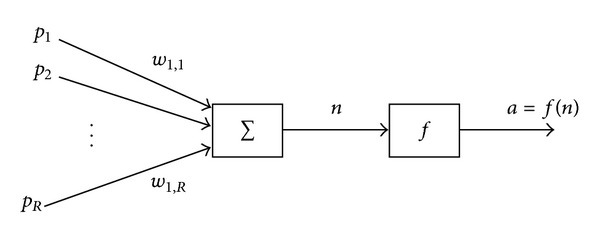
Basic neural model.

Single neuron model (also known as perceptron) is basic neural model in neural network. In this model, it can consist of multiple input and single output. In Figure 3, there are multiple inputs, *p* = (*p*_1_, *p*_2_, …, *p*_*R*_) that sent through connections that carry weight, = (*w*_1,1_, …, *w*_1,*R*_). 

According to [6], weighted sum of input is then calculated by following

(1) $$\begin{array}{c} \langle w,p\rangle =\sum_{i=1}^{n}w_{i}\cdot p_{i}. \end{array}$$

Weighted sum of input is then compared to threshold. In this case, weighted sum of input is compare to an activation function which is Heaviside function, and it is defined as

(2) $$\begin{array}{c} F\left(x\right)=\{\begin{array}{cc} 1 & \text{for}\;x>0\\ 0 & \text{for}\;x\leq 0. \end{array} \end{array}$$

Hence, the overall formula is given by

(3) $$\begin{array}{c} Y=f\left[\langle w,p\rangle \right]=f\left(\sum_{i=1}^{n}w_{i}\cdot p_{i}\right). \end{array}$$

**Figure 4:**
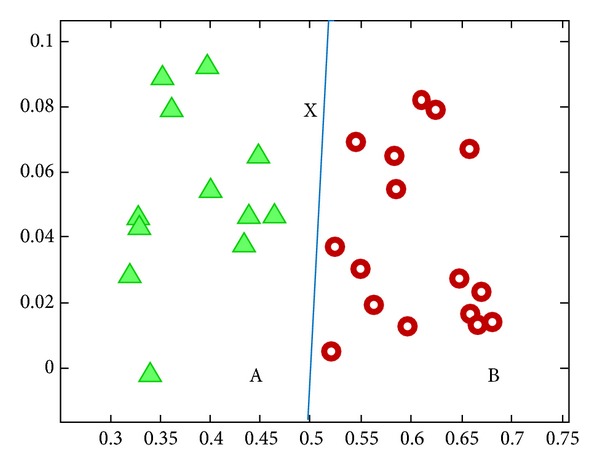
Classification of input generated by perceptron.

From Figure 4, the inputs are belonging to different classes A and B that are separate by the decision boundary. Decision boundary is generated by the perceptron by using the weights. If the boundary does not give correct classification, the weight changes until it gets correct classification.

**Figure 5:**
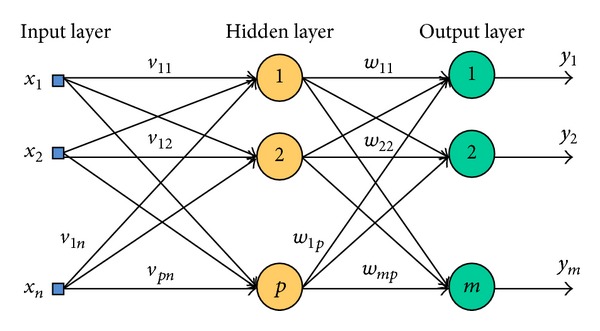
Neural network with one hidden layer of neurons.

In addition, multilayer perceptron generally consists of more than three layers which are comprise of input layer, output layer, and more than one hidden layer. In Figure 5, the hidden layer is located between input layer and output layer. Each node in one layer connects with weight *w*_*ij*_ to every node in the following layer. In multilayer perceptron, it utilized feed forward network with sigmoid transfer function (activation function). The input connection directly goes to hidden layer, but no output layer and the information go in one direction (feed forward).

According to [6], in Figure 5, there are multiple inputs, *x* = (*x*_1_, *x*_2_, …, *x*_3_) and outputs*y* = (*y*_1_, *y*_2_, …, *y*_3_). Weighted sum of input should be calculated by *u* = *f*(*v* · *x*) and defined as

(4) $$\begin{array}{c} \left(\begin{array}{c} u_{1}\\ u_{2}\\ u_{p} \end{array}\right)=f\left(\left(\begin{array}{cccc} v_{01} & v_{02} & \ldots & v_{0n}\\ v_{11} & v_{12} & \ldots & v_{1p}\\ v_{p1} & v_{p2} & \ldots & v_{pn} \end{array}\right)\cdot \left(\begin{array}{c} 1\\ x_{1}\\ x_{n} \end{array}\right)\right). \end{array}$$

Next, the result is used to compute the output by this formula *y* = *f*(*w* · *u*) and defined as

(5) $$\begin{array}{c} \left(\begin{array}{c} y_{1}\\ y_{2}\\ y_{p} \end{array}\right)=f\left(\left(\begin{array}{cccc} w_{01} & w_{02} & \ldots & w_{0n}\\ w_{11} & w_{12} & \ldots & w_{1p}\\ w_{p1} & w_{p2} & \ldots & w_{pn} \end{array}\right)\cdot \left(\begin{array}{c} 1\\ x_{1}\\ x_{n} \end{array}\right)\right). \end{array}$$

Note that *v* = (*v*_01_, *v*_02_, …, *v*_*pn*_) and *w* = (*w*_01_, *w*_02_, …, *w*_*pn*_) are weight that used to calculate the output of *y*. Hence, the input-output mapping can be summarise as *f*(*x*) = *f*(*w* · *y*) = *f*(*w* · *f*(*v* · *x*)) with one hidden layer.

There are two types of neural network methods used to identify disease susceptibility genes which are linkage analysis and association analysis [7]. In linkage analysis, the main focus is to detect linkage between a disease locus and a marker. The testing hypothesis is testing whether a region of gene contains disease susceptibility gene. Generally, the input of neural network is genotypes, whereas the output of neural network is phenotype values (for instance, disease status and quantitative clinical variable such as levels of insulin). There are various types of encoding strategies for inputs and outputs of neural network. For instance, the inputs can be the presence or absence of marker allele in which value 1 indicates the presence of allele and value 0 indicates the absent of allele for each marker in dataset. Besides, the most common encoding strategy for the input of neural network is by using identify-by-descent (IBD). In IBD, variable *x* = 1 is for sharing an allele, *x* = −1 is for not sharing allele and *x* = 0 for uninformative. In contrast, the outputs of neural network also have various encoding strategy. For instance, the output is the disease status in which value 1 = affected whereas value 0 = unaffected.

For association analysis, it has been used to detect linkage disequilibrium between disease locus and marker. The collected data are comprised of genotypes for multiple markers in the sample which is case-control data or cases with family-based controls. Curtis [8] and Curtis et al. [9] had utilized association analysis in their study in which there are four input nodes which is four markers coded as genotypes 0, 1, and 2, whereas the target output is value 1 for cases and value 1 for controls. Hence, the architecture of neural network varies and it should depend on the types of analysis.

### 2.2. Application of Neural Network Method for Detecting Gene-Gene Interactions

Neural network which is pattern recognition method that is used to address the challenges for human geneticists and the explosion of genetic information which eventually leads to exhaustive search of multilocus computationally infeasible. However, the architecture of neural networks is the key of success for detecting gene-gene interactions. Hence, neural networks should evolve the best neural network architecture for particular method. As a result, genetic programming neural network (GPNN) is an evolved methods of optimizing the architecture of a neural network which enhance the identification of genetic combination (or gene-gene interactions) with disease risk [10].

Ritchie et al. [11] had utilized genetic programming to optimize the architecture of neural network (GPNN) and back propagation neural network (BPNN) to model gene-gene interactions. Both of the methods had used simulated data from a set of different model which possess the interaction between genes (can be denoted as epistasis) with the presence of functional single nucleotide polymorphisms (SNPs) and nonfunctional SNPs. First of all, Ritchie et al. [11] construct BPNN by using feed-forward network which consists of one input layer with zero until two hidden layers and one output layer. In addition, GPNN had been used to optimize the variables, weights, and connectivity of the network. The result had showed that GPNN had outperformed BPNN when these two methods are applied to the model which contains both functional and nonfunctional SNPs. This is due to GPNN possess 100% prediction power for all model with greater than 0.026 heritability whereas BPNN only had 80% power for model which had greater than 0.051 heritability value. Noted that the higher the heritability value indicates the stronger genetic effect. Furthermore, GPNN had lower prediction error than BPNN, whereas BPNN had lower classification error. Hence, the authors have concluded that GPNN had improved prediction error and possessed higher power than BPNN. 

In 2004, Tomita et al. [12] had used artificial neural network to predict the development of childhood allergic asthma. To achieve this, artificial neural network was implemented along with parameter decreasing method. This is used to analyse 25 SNPs of 17 genes and select 10 susceptible SNPs among the Japanese people. The result had showed that total 97.7% accuracy was predicted for learning data and total accuracy of 74.4% for evaluation data in 10 SNPs. The authors had concluded that artificial neural network is the most suitable methods to select SNP combinations that are associated with childhood allergic asthma.

In the year of 2005, a method which utilized genetic algorithms combined with neural network components was developed to determine gene interactions in temporal gene expression data sets [13]. This method was able to find the gene network that fit the gene expression data and that is effective on both artificial and real-world expression data on a per-gene basis. Moreover, this method can find gene network in reasonable computational time. This method utilizes genetic algorithms and supervised single-layer artificial neural network in which one individual chromosome of the genetic algorithm can be used to represent a small number of genes from the dataset. Next, artificial neural network is used to determine how well of the input (for e.g., expression values of these genes) at one time points that affect output (for e.g., another gene's expression values) at the subsequent time points over a temporal data points. This method discovers the sets of genes that have the greatest effect in the dataset by selecting the most suitable mutation and crossover operators. The result had shown that this method discovers gene network that can accurately fit on both artificial and real world gene expression data. Furthermore, it also found models from a number of training examples, and this method produces good accuracy by reproducing the test data examples. The authors had concluded that this method found gene network, and it is a multipurpose tool for the application of analysing gene expression data.

GPNN was applied in the studies of human disease to detect gene-gene and gene-environment interactions as pattern recognition method [14]. GPNN was able to select the optimal architecture for feed-forward back propagation neural network, and it is able to improve trial and error process. A GPNN algorithm was able to produce optimal neural network architecture for a given dataset. It does this by optimizing the inputs from a large number of variables, weights, and connectivity of network (e.g., number of hidden layers and the nodes of hidden layers). Hence, GPNN outperformed traditional back propagation neural network due to traditional method only the weights are optimized with prespecified inputs and architecture of neural network. The result showed that GPNN can detect even relatively small genetic effects with 2% to 3% heritability in simulated data model that involve two and three locus interactions. GPNN also reached limit of detections with less than 1% heritability when interactions are more than three loci. The authors have concluded that GPNN is a powerful method in the human disease fields in detecting gene-gene interactions and gene-environment interactions.

Furthermore, research carried out by Ritchie et al. [15] also showed that GPNN has 100% power for heritability estimates of 5% compared with most common disease that have overall heritability estimates greater than 20%. These results show that GPNN has outperformed traditional back propagation neural network, stepwise logistic regression (SLR), and classification and regression trees (CART) to detect gene-gene interactions in models with very small heritability values. Furthermore, GPNN can be used to study complex nonlinear interactions with binary endpoints in any number of disciplines as LR and CART had previously applied. 

Grammatical evolution neural network (GENN) was introduced to detecting gene-gene or gene-environment interactions in high dimensional genetic epidemiological data [16]. It has been used in detect interactions in the presence of noise. Noise occurs are often caused by genetic heterogeneity, genotyping error, phenocopy, and missing data. GENN has shown highly success in the range of simulated data. This research had shown that GENN is highly robust towards genotyping error and missing data. Moreover, GENN has higher success than multifactor dimensionality reduction (MDR) when GENN is present to detect functional loci in the presence of genetic heterogeneity. GENN had outperformed that MDR may be because it utilizes evolutionary search strategy and Boolean operator is used in the grammar. However, the power of GENN reduces due to the presence in phenocopy in dataset.

Günther et al. [17] had used neural network to model various types of two-locus disease model. To achieve this, six neural networks (feed-forward multilayer perceptron) with five hidden neurons were carried out with 100 datasets that are generated for each of six two-locus disease models. These models are considered in both high and low risk scenario. Moreover, there are two kinds of models which are multiplicative and epistasis model. The result had showed that neural networks outperformed logistic regression and multifactor dimensionality reduction in modelling the biological interaction of the disease model. Neural network generally had lower mean absolute differences between the estimated penetrance matrices and the theoretical penetrance matrices if compared with logistic regression and multifactor dimensionality reduction. The authors had demonstrated that neural networks are the promising tool to handle the complex disease data.

Turner et al. [18] had proposed a stochastic method to discover genetic mechanisms that can affect human trait for the disease processes by detecting and modelling gene-gene and gene-environment interactions. Generally, ATHENA is denoted as the analysis tool for heritable and environment network associations. This method had utilized alternative tree based crossover, back propagation for locally fitting neural network weights. In addition, this method also obtains the domain knowledge from biological databases and incorporates it into the method so that the search for gene-gene interaction was initialized. By applying simulated dataset, this method had shown the modest increase in the sensitivity. Furthermore, it also found the highly statically significant increase when back propagation was used to fit the neural network weights locally when the search space is larger than search coverage. At last, authors had concluded that this hybrid optimization method can increase the sensitivity and performance for detecting and modelling gene-gene interactions that can bring huge impact on the complex human trait.

 Hardison and Motsinger-Reif [4] had apply GENN to quantitative traits (QTGENN) to a range of simulated genetic models due to the increasing use of GENN in a range of simulated real case control studies. QTGENN is used to detect quantitative trait association with a broad range of effect sizes. As mentioned above, GENN allows grammar modification which contains symbols for arithmetic operators and it allows QTGENN to evaluate quantitative traits with GENN. QTGENN also have the input variables that can read input data for each sample that consists of genotypes and potential environmental covariates. The results had shown that QTGENN method has high power than traditional linear regression analysis methods to detect single-locus model and completely epistatic two-locus models.  Table 1 summarizes the researches that used neural network method to detect and model the gene-gene interactions.

## 3. Support Vector Machine

According to [19], support vector machine (SVM) is machine learning algorithm that is utilizing hyperplanes in high dimensional plane and is often used in classification and regression tasks. SVM does its task by analyses data and recognizes that data's patterns.

### 3.1. Methodology of Support Vector Machine

According to [20], linear SVM classified the training vector for linearly separable data. Let us assume the training vector, which is defined by

(6) $$\begin{array}{c} D={\left\{\left(x_{i},y_{i}\right)\;|\;x_{i}\in {\mathbb{R}}^{P},y_{i}\in \left\{-1,1\right\}\right\}}_{i=1}^{n}. \end{array}$$

In this case, *x*_*i*_ is denoted as *p*-dimensional vector that is waiting to be classified into *y*_*i*_ implies that there are two classes (for −1 and 1). 

**Figure 6:**
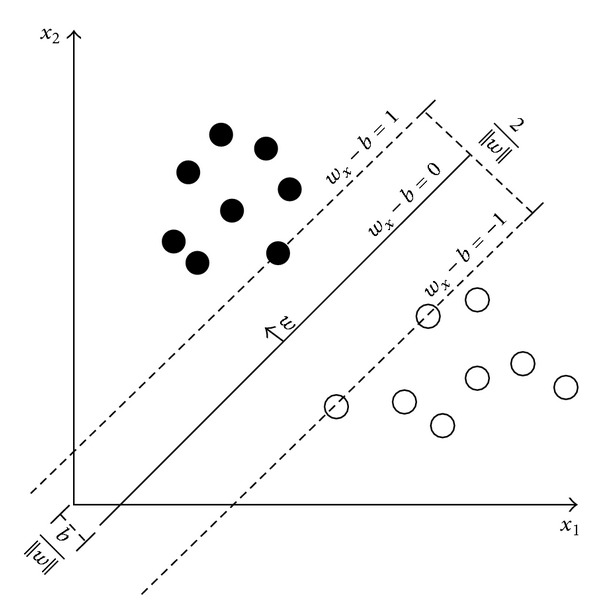
Linear SVM with maximum-margin hyperplane.

In Figure 6, a hyperplane separates two classes of input that satisfying points *x* is given by *w* · *x* − *b* = 0 where *w* is the normal vector, *b* is the bias, and “·” is the dot product. According to [20], there are additional two hyperplane that separate the data from the previous hyperplane (defined by *w* · *x*_*i*_ − *b* = 0) where there is no data between them. The additional hyperplane consists of maximum distance to the hyperplane (known as margin) that can prevent data points from falling into the margin and is defined by *w* · *x*_*i*_ − *b* ≥ 1 for all *x*_*i*_ that classified in first class and *w* · *x*_*i*_ − *b* ≤ 1 for all *x*_*i*_ that classified in second class.

Furthermore, in Figure 6, the distance between two hyperplane is calculated by 2/||*w*||, and the offset of the hyperplane from the origin along the normal vector *w* is determine by *b*/||*w*||. Overall equation for the additional hyperplane can be written as

(7) $$\begin{array}{c} y_{i}\left(w\cdot x_{i}-b\right)\geq 1,\;\forall 1\leq i\leq n. \end{array}$$

There are some data that are difficult to separate because the data are too “noisy.” Hence, the input vector data are mapped into higher dimensional space which is feature space so that the linear separation is achieved.

Missiuro [21] had made an assumption that input argument (*x*_*i*_, *x*_*j*_) is expressed into inner product (*φ*(*x*_*i*_), *φ*(*x*_*j*_)) where kernel is a function that is defined as

(8) $$\begin{array}{c} K\left(x_{i},x_{j}\right)=\left[\varphi \left(x_{i}\right),\varphi \left(x_{j}\right)\right], \end{array}$$

where *φ* is kernel function that map input space into feature space. 

**Figure 7:**
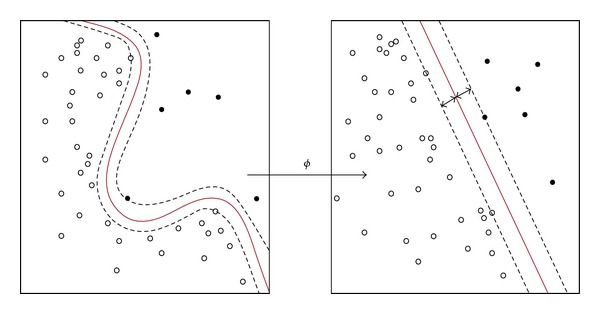
Input space are mapping into feature space by using kernels method.

Kernel firstly maps the data into a feature space. After that, Kernel performs linear classification in the feature space. In Figure 7, linear separation is achieved when input space are mapping in feature space by using kernels method. According to [22], the examples of kernels are linear, polynomial, and radial basis function.

According to [21], SVM can be used to predict which genes are genetically interacting with each other by learning from the features which are known genetically interacting pairs. To achieve this, the training data of SVM are the two sets of feature vectors which are label as positive (indicates presence of genetic interaction) and negative (lack of genetic interaction). For each features vector, it characterizes a pair of genes. These features are mapped into high dimensional space by separating into genetically interacting pairs and nongenetically pairs using hyperplane that possesses maximum margin. This mapping is done by using kernel function such as polynomial or radial basis function kernel. 

### 3.2. Application of Support Vector Machine Method for Detecting Gene-Gene Interactions

Matchenko-Shimko and Dubé [23] had utilized both SVM and artificial neural network (ANN) to investigate gene-gene and gene-environment interactions as determinants in complex disease. They had extended the SVM and ANN regression models with preselection of SNP-SNP combination and this is to test the important of potential interactions between genes. The results had shown that both preselection strategies performed well in various types of parameters and models except for those dataset with low marginal effects and combination of low disease allele frequencies. In addition, there exist some parameters to determine the power of ANN and SVM in detecting interacting SNPs. The parameters are included marginal effect size of disease loci, allele frequency of the disease loci and linkage disequilibrium between disease and marker loci, and the sample size. At last, the authors concluded that larger sample sizes are needed to determine gene-gene interaction involving SNPs with low marginal effect sizes compared with interaction with moderate marginal gene effect sizes. Furthermore, both machine learning methods had well performed in increasing allele frequency with low linkage disequilibrium and/or low marginal gene effects.

Chen et al. [19] had apply SVM in various kinds of combinatorial optimization methods (for e.g., local search and genetic algorithm) which is recursive feature addition (SVM-RFA), recursive feature elimination (SVM-RFE), local search (SVM-Local), and genetic algorithm (SVM-GA) to detect gene-gene interactions. This paper tends to review the data mining approach that deals with binary trait outcomes (for e.g., disease status) that have been accepted to detect interactions between genes. The result had showed that both SVM-Local and SVM-GA able to achieve good results when dealing with unbalanced data except in dealing with five single-nucleotide polymorphisms (SNPs) combinations. However, MDR outperformed SVM in dealing with balanced data. In addition, SVM methods had achieve better results by considered all case, control, and average accuracies. The methods are able to maximize the average accuracy and it also can balance the case-control accuracy. The author concluded that SVM methods were able to discover the best candidate models, and it is more reliable compared with MDR. Furthermore, the methods suggest by the author had less concentration on overfitting, being able to handle unbalanced data and discover more stable models. 

Özgür et al. [24] had proposed an automatic method that utilized automatic literature mining and network analysis to extract known and infer unknown gene-disease association. Firstly, initial set of known disease related genes are collected, and interaction network was built by automatic literature mining based on dependency parsing and SVM. This research assumes that central genes are likely to be related to the disease. The criteria such as degree, eigenvector, betweenness, and closeness centrality metrics are considered to rank the genes in the network. This method was evaluated by using prostate cancer, and it achieved high accuracy for eigenvector and degree centrality. The result of this research showed that the total of 95% of top 20 genes ranked are confirmed to be related to prostate cancer. However, betweenness and closeness centrality metrics predicted more genes which are related to the disease that is currently unknown and that candidates for future experimental study. The authors concluded that the proposed method was an approach that can be utilized to extract known gene-disease associations from the literature, and it is also able to infer unknown genes to be a candidate for future experimental analysis. 

Shen et al. [25] had proposed two stage methods to detect gene-gene interactions. Firstly, SVM with L1 penalty (a model selection method) was used to identify the most promising SNPs and interactions. The second stage was to the application of logistics regression and ensuring a valid type 1 error was achieved by excluding non-significant candidates after Bonferroni correction. The result had shown that SVM with L1 penalty was useful in practical methods for case-control association studies in which multiple logistic regression perform is better than traditional logistic regression for each identified SNP or interaction.

In Ban et al. [26], SVM method had been applied to analyse the interactions between genes in type 2 diabetes mellitus (T2D) by examining 408 SNPs in 87 genes are that involved in major T2D related pathway. The pathways involved 426 T2D patients and 456 healthy controls from the studies of Korean cohort. The results of the studies show that SVM with radial basis function (RBF) was applied in combination of 14 SNPs in 12 genes had achieved 65.3% prediction rate. Moreover, this method also had been achieved the prediction accuracy with 70.9% and 70.6% when being applied in subpopulation datasets of men and women and identified different SNP combination. The authors had drawn conclusion that a novel association between combinations of SNPs and T2D in a Korean population can be achieved by using support vector machine based feature selection method. 

Missiuro [21] had used SVM to detect genetic interactions in kinase families of genes in *Caenorhabditis elegans*. Firstly, collaborative filtering (CF) is used to fill the missing entries input data matrix for SVM. In addition, individual gene features or CF-reduced form of gene features are merged together as the input for SVM. Next, various kinds of kernels include linear, polynomial of degree two until five, and radial basis function was implemented along with SVM. The cross validation result showed that RBF kernel was more effective in predicting genetic interaction if compared to CF. The predictive accuracy can also increase when the author narrow down the genes to specific functional category which is kinase families of genes.

Fang and Chiu [27] had proposed extended SVM and SVM based pedigree-based generalized multifactor dimensionality (PGMDR) to study the interactions between genes. This study was using limited samples of families to study the interactions in the presence (or absence) of main effects of genes with an adjustment of covariates. The result showed that proposed extended SVM and SVM-based PGMDR have high accuracy compared to PGMDR and FAM-MDR (family-based multifactor dimensionality reduction). Moreover, the extended SVM method has the highest prediction accuracy, but it possesses low consistencies if compared to SVM-based PGMDR method. The results indicated proposed SVM method can be used for prediction for both simulation study and data example. Furthermore, SVM-based PGMDR can be used to discover the genetic model. The authors concluded the proposed methods are particularly useful in detecting gene-gene and gene-covariate interactions in limited samples of families.

Zhang et al. [28] had implement SVM along with binary matrix shuffling filter (BMSF) to classify cancer tissue samples from gene expression data by taken account the possible interactions among genes. By integrating BMSF in SVM, it solves the problem associated with search schemes of traditional wrapper method and overfitting. In addition, it also selects potential gene interaction during gene selection. To achieve this, the set of genes are kept into SVM model which was recursively refined and updated. It depends on the effect of a gene contributing to the other genes to refer the usefulness of the genes during cancer classification. The result from the research had shown that the average accuracy of 91.19% and 97.69% was achieved across nine dataset. Furthermore, BMSF also had been applied in Linear Discriminant Analysis (BMSF-LDA) and Quadratic Discriminant Analysis (BMSF-QDA) with average accuracy which is 94.82% and 94.67%, respectively across nine dataset. The authors had concluded that efficient search scheme (BMSF) in SVM can increase the efficiency search by including possible interactions of many genes.

Marvel and Motsinger-Reif [29] had used SVM with grammatical evolution (GE) as a method to select features and parameters (GESVM). GE is particularly important to select features, parameters or kernels that should be included in SVM. The combination of GE with SVM was used to identify gene-gene and gene-environment interactions in complex human disease especially on large dataset. GE was particularly important to select feature in large dataset as many traditional methods had failed to be applied. The result of the studied had shown that the SVM is able to discover SNP_1_ and SNP_2_ for disease model M_1_ with 73.3% expected accuracy. Moreover, the method is able to identify SNP_2_ for disease model M_2_ with 58.3% expected accuracy. The authors also stated that the future work should focus on performing parameter sweeps for GE and SVM parameters for different dataset to discover the true potential of GESVM.  Table 2 summarizes the researches that used SVM method to detect and model the gene-gene interactions.

## 4. Random Forest

According to [30], random forest (RF) is a collection of individual decision-tree classifiers. A bootstrap sample of instances from the data is used to train each tree in the forest. Then, choose each split attribute in the tree from a random subset of attributes. Classification of instances is based upon aggregate voting over all trees in the forest.

### 4.1. Methodology of Random Forest

All trees of RF are frown to their full extent without pruning because each tree of a RF is grown using random feature selection to select a training set (bootstrap sample) from the original data [31]. Based on [32], a classifiers decision tree of RF is grown as follows.

1. Select two-third of original data for training. The selected training data is defined as size *N* which is a total number of trees to grow (*ntree*) bootstrap samples.
2. The left data from original data are called the “out-of-bag” (OOB).
3. Grow an unpruned classification or regression tree (CART) for each bootstrap sample as below.
  - At each node in the tree, the total *p* predictor variables are randomly select variables of pernode (*mtry*).
  - Maximize some measure of node purity from among the mtry variables, such as Gini index to choose the best split at each node.
  - Use the OOB individuals to obtain an estimate of prediction error for each tree.
4. The OOB is observed to determine the final prediction/classification over all trees. Accuracy of the OOB prediction over all subjects is calculated to consider the OOB prediction error and prediction accuracy of the RF.

### 4.2. Application of Random Forest Method for Detecting Gene-Gene Interactions

The RF method was used by Lunetta et al. [33] as a screening procedure to identify a set of risk-associated single nucleotide polymorphisms (SNPs) from the large number of unassociated SNPs of complex disease models. The result had shown that the improvement on performance of RF analysis relative to Fisher exact test for screening is exponential to the number of interacting SNPs also is increase. The authors had concluded that the RF analyses are better than standard univariate screening methods. This is due to the fact that RF analyses can significantly reduce the number of SNPs from large-scale genetic association. The large-scale genetic association consists of unknown interactions that exist among true risk-associated SNPs or SNPs and environmental covariates.

The RF method used by Jiang et al. [34] is to convert epistatic interactions from a large number of all possible combinations of genetics variants into a manageable set of candidates by reducing the search space for epistatic interactions. The result had shown that SNP markers as categorical features and adopting the RF to discriminate cases against controls are more precisely. The authors concluded that the Gini importance of RF was offering another measure for the associations between SNPs and complex diseases. This complement the existing statistical measures used to understand the epistasis in the pathogenesis of complex diseases and identify the epistatic interactions.

A new method of RF which is random jungle (RJ) which has been proposed by Schwarz et al. [35] using permutation importance measures to detect important single nucleotide polymorphisms. RJ is able simultaneously to perform RF by using parallel techniques which are multicomputer and multithreading. Thus, RJ is better than RF. From the result, RJ can maintain all options in other programs and had a new function which is backward elimination method. RJ is also able to compute up to 159 times faster than the fastest alternative implementation. The authors illustrate the most important SNPs validate recent findings in the literature and reveal potential interactions by the application of RJ to a genome-wide association of Crohn's disease.

The method of RF used by Liu et al. [36] is to identify rheumatoid arthritis (RA) susceptibility by contributed gene-gene interactions and to identify SNPs which can distinguish who were anticyclic citrullinated protein positive and healthy controls among RA patients. The result showed that RF had distinguish RA cases from controls with 70% accuracy when applied to a set of SNPs selected from single-SNP and pairwise interaction tests identified 93 SNPs. The authors concluded that the most classification information provided by HLA SNPs nevertheless non-HLA SNPs improved classification. A stepwise method of combining association and classification methods is needed. This is able to distinguish RA cases from healthy controls by identifing candidate interacting SNPs. Hence, this new method can overcome the difficulty to validate specific gene-gene interactions using genome-wide SNP data.

The RF method used by Winham et al. [32] is to identify gene-gene interactions that have not been thoroughly explored and used to detect high-dimensional gene-gene interaction effects and their potential effectiveness. The result shows that RF is effectively in low dimensional data to identify gene-gene interactions. The authors concluded RF is still considered as a promising data-mining technique. This is because RF can simultaneously extend univariate methods to multiple variables condition. Besides, RF might not suitable as a filter technique because RF variable importance measures fall to detect interaction effects in high-dimensional data in the absence of a strong marginal component. 

The mutual information network (MIN) integrated with RF method is called mutual information network guided random forest (MINGRF). This proposed method proposed by Pan et al. [37] is to reduce bias of RF towards the marginal main effects and to avoid random sampling for variables. The authors concluded that MINRF was better than RF since the result showed that the MINGRF is more accurate and has shorter computational time. 

The method of RF used by Staiano et al. [38] is to identify the polymorphisms of gene that causes familial combined hyperlipidemia (FCH). The result of the role that identified gene in the development of FCH phenotype is promising and encouraging for further investigation. The authors suggested to characterize patients and to identify high-risk subjects with better using the combined study of different genetic variations.

The proposed new hunting pathway using random survival forests (RSF) method was used by Chen and Ishwaran [39] to account for important pathway of gene correlation and genomic interactions. The result indicates that signalling pathways can be identified in a relatively small sample size using the RSF pathway hunting algorithm. The authors concluded that the proposed algorithm is an efficient and powerful framework to model pathway from a high-dimensional genomic data.  Table 3 summarizes the researches that used random forest methods to detect and model the gene-gene interactions.

## 5. Discussion and Conclusion

This paper had presented the machine learning methods that is used to detect gene-gene and gene-environment interactions in common and complex human multifactorial disease. Those methods are neural networks (NNs), support vector machine (SVM), and random forests (RFs). Each method has its own strengths and weakness in dealing with such epitasis model.  Table 4 summarizes the strengths and weaknesses of neural networks, support vector machine, and random forests methods for detecting gene-gene interactions.

As previously mentioned, the greatest challenges in detecting gene-gene interactions is the increasing volume of data due to the increasing number of SNPs that will increase the potential interactions among genes. The advent of machine learning methods can address these challenges. Among this methods, neural networks are able to address the problem found in gene-gene interactions especially on the case focusing on genetic heterogeneity, polygenic inheritance, high phenocopy rates, and incomplete penetrance [31]. Moreover, the ability of neural network in unraveling the pattern on given dataset and hidden potential rules does not overfitting the data and had make neural network efficiently detecting more complex interactions among genes. The optimal architecture of neural network can also be optimized by using evolutionary algorithm in finding nonfunctional SNPs. For example, GPNN and GENN are capable to show their robustness on examining high dimensional and noise datasets.

Support vector machine is a powerful method in detecting gene-gene interactions in both real and simulated dataset. It is due to its capability on classifying the samples by using linear and nonlinear separators. Linear separator is able to classify the data into two classes, whereas nonlinear separator such as quadratic kernel can be used to classify the sample by modifing the input space. Moreover, this method is similar with neural network in which it also does not overfitting the data and robust to noise. 

Besides, random forests is a natural method to detect interactions among genes. This method is particularly useful in solving the case of genetic heterogeneity because the subset of the model are separated in the early stage. Thus, it showed high performance in detecting gene-gene interaction in diverse tree model. It does not overfitting the data, and hence it does not need to prune the tree structure. Random forests is also readily to be used in attribute interaction that motivates it as a competitive method to discover the gene-gene interactions. 

Currently, there are no best machine learning methods that can be best in detecting gene-gene interactions by involving various types of dataset and problems found in detecting gene-gene interactions. To address this challenges, several machine learning methods can be integrate to form a framework that can efficiently detect interactions among genes. For instances, two-stage model can be constructed from the machine learning methods in order to exhaustively detect the gene-gene interactions. The resulted model may be easy to interpret and take the shortest time to detect gene-gene interaction.

Furthermore, data mining methods such as multifactor dimensionality reduction (MDR) can be introduced to examine the gene-gene interactions in human disease because it can taking account every possible combinations of variables in a given order. It will eventually solve the problems that the imbalance between experimental output and theoretical understanding in unexplained experimental data in genetics case study. In addition, MDR also possesses the ability on analysing the quantitative traits that makes it as a favour method to detect interactions among genes. Hence, future research can focus on data mining methods in order to understand the biological mechanism of the human disease. 

In conclusion, future research on producing more powerful machine learning methods is required to handle the enormous data in order to understand the genetic epidemiology of human disease. Thus, the output of this machine learning methods can enhance the understanding of the biological mechanism of human disease, and this knowledge can contribute in predicting the clinical disease. 

## Figures and Tables

**Table 1:** Summary of detect gene-gene interaction using neural network method.

No.	Author	Dataset	Description
(1)	Ritchie et al. [11]	Epistasis model.	GPNN and BPNN were used to model gene-gene interactions by using simulated data. The simulated data contains functional SNPs and nonfunctional SNPs which model the interaction between genes.

(2)	Tomita et al. [12]	Childhood allergic asthma (CAA).	Artificial neural network was utilized with parameter decreasing method in order to analyse susceptible SNPs among the Japanese people.

(3)	Keedwell and Narayanan [13]	Artificial data experiments, rat spinal cord and yeast* Saccharomyces Cerevisiae* cell cycle.	Genetic algorithm which was implemented along with neural networks discovers gene-gene interactions in temporal gene expression dataset by elucidating the information between regulatory connections and interactions between genes, proteins, and other gene products.

(4)	Motsinger et al. [14]	Parkinson's disease.	GPNN had been used to optimize the architecture of neural network. This method can be used to enhance the identification of gene combinations associated with Parkinson's disease.

(5)	Ritchie et al. [15]	Alzheimer's disease, breast's disease, colorectal disease, and prostate's disease.	GPNN had been used to detect gene-gene interactions and gene-environment interaction in studies of human disease to optimize the architecture of Neural Network by using simulated dataset.

(6)	Motsinger-Reif et al. [16]	Epitasis model.	GENN was utilized to discover gene-gene interactions that caused are by noise (for instance, genotyping error, missing data, phenocopy, and genetic heterogeneity) in high dimensional genetic epidemiological data.

(7)	Günther et al. [17]	Two-locus disease models, multiplicative and epistasis model.	NN had been used in simulation study to model the different kind of two-locus disease model by constructing six neural networks.

(8)	Turner et al. [18]	Simulated human.	ATHENA had been used to discover the gene-gene interactions that influence complex human traits by integrating alternative tree-based crossover, back propagation, and domain knowledge in ATHENA.

(9)	Hardison and Motsinger-Reif [4]	Genetic models.	QTGENN had applied GENN methods to quantitative traits in various types of simulated genetic models. This method had been successfully applied in single-locus models and two-locus models.

**Table 2:** Summary of detect gene-gene interaction using support vector machine method.

No.	Author	Dataset	Description
(1)	Matchenko-Shimko and Dubé [23]	Simulated disease.	Both SVM and artificial neural network (ANN) were used to preselect the combination of SNP to test the importance of potential interactions between genes in complex disease.

(2)	Chen et al. [19]	Real prostate cancer genotyping.	SVM was applied in different kinds of combinatorial optimization methods which were recursive feature addition, recursive feature elimination, local search, and genetic algorithm.

(3)	Özgür et al. [24]	Prostate cancer.	Automatic method that was proposed to extract known genes-disease and infer unknown gene-disease association by using automatic literature mining based on dependency parsing and support vector machines.

(4)	Shen et al. [25]	Parkinson disease.	Authors had employ two-stage method by using SVM with L1 penalty to detect gene-gene interactions for human complex disease.

(5)	Ban et al. [26]	Type 2 diabetes mellitus-related genes.	SVM was used to predict the importance of gene-gene interactions in T2D in the studies of Korean cohort studies.

(6)	Missiuro [21]	*Caenorhabditis elegans*.	SVM was utilized in this research to detect interactions between gene in kinase families for *Caenorhabditis elegans *organism*. *

(7)	Fang and Chiu [27]	COGA (genetics of alcoholism).	SVM-based PGMDR was introduced to study the interactions of gene-gene and gene-covariate in the presence or absence of main effects of genes.

(8)	Zhang et al. [28]	Human cancer.	Binary matrix shuffling filter (BMSF) as an efficient SVM search schemes was integrated with SVM to classify cancer tissue samples.

(9)	Marvel and Motsinger-Reif [29]	Disease model, M_1_ and M_2_.	GESVM was applied in large dataset to select important features, parameters, or kernel in SVM.

**Table 3:** Summary of detect gene-gene interaction using random forest method.

No.	Author	Dataset	Description
(1)	Lunetta et al. [33]	H2M2, H4M2, H8M2, H16M2, H4M4, and H8M4.	RF as a screening procedure to identify top-ranked true-associated SNPs which can cause disease without losing any interactions.

(2)	Jiang et al. [34]	Three simulated disease model.	RF is used to recognize the cases that were against controls and to obtain the Gini importance which is used to measure the contribution of each SNP to the classification performance.

(3)	Schwarz et al. [35]	Crohn's disease.	A new method of RJ based on basis RF knowledge was developed to facilitate a fast processing in the high-dimensional of genome-wide analysis data of gene-gene interactions.

(4)	Liu et al. [36]	NARAC1 and NARAC2.	RF is used to detect contributed gene-gene interactions for identifing RA susceptibility and to identify SNPs of RA patients to classify them into anticyclic citrullinated protein positive and healthy controls.

(5)	Winham et al. [32]	Five models.	Focus on identifing rarely gene-gene interactions and detecting gene-gene interaction effects and their potential effectiveness on high-dimensional data using RF.

(6)	Pan et al. [37]	Bladder cancer.	The proposed method of MINGRF is proposed to improve the performance of RF such as accuracy and computational time.

(7)	Staiano et al. [38]	Familial combined hyperlipidemia (FCH).	RF is used to identify gene-gene interactions that are involved in FCH. FCH increase the plasma triglycerides and/or total cholesterol level of patients and hence increase the risk of coronary heart disease.

(8)	Chen and Ishwaran [39]	Colon cancer and ovarian cancer.	RSF as new hunting pathway to detect gene correlation and genomic interactions from a high-dimensional genomic data.

**Table 4:** Strengths and weaknesses of neural networks, support vector machine, and random forests methods for detect gene-gene interactions.

Methods	Author	Strengths	Weaknesses
Neural network	Musani et al. [40] Upstill-Goddard et al. [31]	(i) NN is able to model the relationship between disease and single nucleotide polymorphism (SNP) (ii) NN can make prediction on data where the disease outcome is unknown by learning the outcome given on a dataset (iii) NN is a method that can deal with large volumes of data (iv) NN is suitable for genetic heterogeneity, high phenocopy rates, polygenic inheritance, and incomplete penetrance. (v) GPNN and GENN are able to optimize the architecture of NN and possess high power to discover the presence of nonfunctional SNPs. (vi) GPNN does not overfitting the data (vii) GPNN possesses high power in dealing with epitasis model with weak marginal effect (viii) GENN outperform GPNN by optimiz NN in fewer generations (ix) GENN possesses high power to detect high risk loci in complex disease	(i) Presence of black box (ii) Difficult to list out all possible NN architecture and it causes the difficulty to find the optimal architecture (iii) GPNN needed parallel processing environment (iv) GPNN causes the high false positive rate to occur in three locus models (v) The output of GPNN is binary expression, and it can be hard to interpret (for instance, up to 500 nodes) (vi) Result of NN was hard to interpret due to the dimensionality problem (vii) NN needs comprehensive cross-validation to confirm validity

Support vector machine (SVM)	Chen et al. [19] Wasan et al. [41] Upstill-Goddard et al. [31]	(i) SVM can deal with high dimension data set (ii) SVM can be utilized to classify complex biological gene expression data (iii) Does not trap at local minima (iv) Not prone to overfitting (v) SVM is robust to noise (vi) The output of SVM is more interpretable if compared to MDR (vii) Does not require user-defined decisions for classification (viii) SVM is ready to be generalized to new structures	(i) Presence of black box (ii) SVM is restricted to pairwise classification (iii) SVM cannot be directly used for feature selection (iv) Result produced may be affected by the presence of missing data (v) The power of SVM might reduce with the presence of genetic heterogeneity (vi) Additional training maybe needed to correct the bias of prediction accuracy. However, it could be computationally expensive for the proposed procedure (vii) Accuracy produced by SVM might be suboptimal due to the SVM parameter C is forced to be one constant. Hence, a grid search for the parameter is needed by utilizing some promising SNP combinations in order to refine the results.

Random forest (RF)	Upstill-Goddard et al. [31]	(i) RF does not exhibit strong main effects which uncover interactions among genes. (ii) RF does not “overfit” the data. (iii) SNPs predictive of a phenotype are identifying by RF.	(i) Presence of black box (ii) RF does not succeed in GWAS data. (iii) Sometimes RF is underestimating important scores of SNPs without marginal effects. (iv) RF only detects interactions with large effect size.
Random jungle (RJ)		(i) RJ is able to analyze data on a genome-wide scale. (ii) RJ has more computationally efficient than RF.	If the main effects are weak, RJ fails to detect interactions.
